# Hedgehog inhibitor sonidegib potentiates ^177^Lu-octreotate therapy of GOT1 human small intestine neuroendocrine tumors in nude mice

**DOI:** 10.1186/s12885-017-3524-x

**Published:** 2017-08-08

**Authors:** Johan Spetz, Britta Langen, Nils Rudqvist, Toshima Z. Parris, Khalil Helou, Ola Nilsson, Eva Forssell-Aronsson

**Affiliations:** 1 Department of Radiation Physics, Institute of Clinical Sciences, Sahlgrenska Cancer Center, Sahlgrenska Academy, University of Gothenburg, Sahlgrenska University Hospital, 413 45 Gothenburg, SE Sweden; 2 Department of Oncology, Institute of Clinical Sciences, Sahlgrenska Cancer Center, Sahlgrenska Academy, University of Gothenburg, Sahlgrenska University Hospital, 413 45 Gothenburg, SE Sweden; 3 Department of Pathology, Institute of Biomedicine, Sahlgrenska Cancer Center, Sahlgrenska Academy, University of Gothenburg, Sahlgrenska University Hospital, 413 45 Gothenburg, SE Sweden

**Keywords:** Radionuclide therapy, radiation biology, Odomzo, LDE225, ^177^Lu-DOTATATE, GEPNET, midgut carcinoid, radiogenomics, radiosensitizer, PRRT

## Abstract

**Background:**

^177^Lu-octreotate can be used to treat somatostatin receptor expressing neuroendocrine tumors. It is highly effective in animal models, but clinical studies have so far only demonstrated low cure rates. Hedgehog inhibitors have shown therapeutic effect as monotherapy in neuroendocrine tumor model systems and might be one option to enhance the efficacy of ^177^Lu-octreotate therapy. The aim of this study was to determine the therapeutic effect of combination therapy using ^177^Lu-octreotate and the Hedgehog signaling pathway inhibitor sonidegib.

**Methods:**

GOT1-bearing BALB/c nude mice were treated with either sonidegib (80 mg/kg twice a week via oral gavage), a single injection of 30 MBq ^177^Lu-octreotate i.v., or a combination of both. Untreated animals served as controls. Tumor size was measured twice-weekly using calipers. The animals were killed 41 d after injection followed by excision of the tumors. Total RNA was extracted from each tumor sample and then subjected to gene expression analysis. Gene expression patterns were compared with those of untreated controls using Nexus Expression 3.0, IPA and Gene Ontology terms. Western blot was carried out on total protein extracted from the tumor samples to analyze activation-states of the Hh and PI3K/AKT/mTOR pathways.

**Results:**

Sonidegib monotherapy resulted in inhibition of tumor growth, while a significant reduction in mean tumor volume was observed after ^177^Lu-octreotate monotherapy and combination therapy. Time to progression was prolonged in the combination therapy group compared with ^177^Lu-octreotate monotherapy. Gene expression analysis revealed a more pronounced response following combination therapy compared with both monotherapies, regarding the number of regulated genes and biological processes. Several cancer-related signaling pathways (i.e. Wnt/β-catenin, PI3K/AKT/mTOR, G-protein coupled receptor, and Notch) were affected by the combination therapy, but not by either monotherapy. Protein expression analysis revealed an activation of the Hh- and PI3K/AKT/mTOR pathways in tumors exposed to ^177^Lu-octreotate monotherapy and combination therapy.

**Conclusions:**

A comparative analysis of the different treatment groups showed that combination therapy using sonidegib and ^177^Lu-octreotate could be beneficial to patients with neuroendocrine tumors. Gene expression analysis revealed a functional interaction between sonidegib and ^177^Lu-octreotate, i.e. several cancer-related signaling pathways were modulated that were not affected by either monotherapy. Protein expression analysis indicated a possible PI3K/AKT/mTOR-dependent activation of the Hh pathway, independent of SMO.

**Electronic supplementary material:**

The online version of this article (doi:10.1186/s12885-017-3524-x) contains supplementary material, which is available to authorized users.

## Background

Neuroendocrine tumors (NETs) are the most common malignancies of the small intestine, and incidence rates are increasing [1, 2]. NETs are a heterogeneous group of malignant neoplasms frequently associated with the synthesis and secretion of peptides and amines causing hormone overproduction symptoms (e.g. carcinoid syndrome). However, NETs are slow-proliferating tumors, and symptoms are seldom evident until at a relatively late stage [3]. Surgery is currently the only curative treatment option for patients with localized NET. However, palliative treatment of NET metastases can be achieved by administration of somatostatin analogs to patients having tumors with high expression of somatostatin receptors (SSTR) [3]. Peptide receptor radionuclide therapy (PRRT) with radiolabeled somatostatin analogue ^177^Lu-[DOTA^0^, Tyr^3^]-octreotate (^177^Lu-octreotate or ^177^Lu-DOTATATE) is another therapeutic option for patients with SSTR-expressing tumors. This treatment has shown successful results regarding tolerability, tumor regression, increased overall survival, and improved quality of life in patients with inoperable disease [4–7]. However, the treatment is limited by the risk organs bone marrow and kidneys, which restrict the amount of ^177^Lu-octreotate administered to the patients. Complete tumor remission is rare and attempts to increase treatment effects using ^177^Lu-octreotate in combination with other systemic treatments (limited by different risk organs), have been performed, with varying success rates [8–10].

The Hedgehog (Hh) pathway is a major developmental signaling pathway, which regulates both proliferation and differentiation of various types of stem cells during embryogenesis [11]. In the absence of Hh ligands, the Hh receptor Patched inhibits activity of the transmembrane protein Smoothened (SMO) [12]. Binding of Hh ligand to Patched results in accumulation of SMO in the primary cilium and activation of transcription factors GLI1, GLI2 (activators) and GLI3 (repressor) [12]. When activated, the GLI proteins translocate into the nucleus and regulate transcription of genes involved in, e.g. cell cycle regulation, cell adhesion, signal transduction, angiogenesis, and apoptosis [13, 14]. Defective Hh signaling has been implicated in various types of human cancers [15], and several components of the Hh pathway have been studied and proposed as targets for cancer treatment [12–14, 16]. Hh signaling has been shown to be activated in NETs, and treatment with Hh inhibitors have resulted in reduced cell viability in vitro [17–19]. Since the Hh pathway is important in cancer initiation and development, it may also be important for tumor radioresistance and regrowth after treatment with ionizing radiation. Preclinically, Hh signaling has been shown to promote radiation resistance, and increased anti-tumor effects have been observed when combining ionizing radiation and Hh inhibitors [2, 13, 20]. Sonidegib (also known as Odomzo®, erismodegib or NVP-LDE225) is a selective and orally bioavailable antagonist of SMO [21], which has previously shown an anti-tumor effect in neuroendocrine tumor models [22]. It has received FDA approval for treatment of basal cell carcinoma, and is currently being investigated as a potential treatment for various cancers (e.g. small cell lung cancer) [23]. Sonidegib treatment is generally well tolerated, but doses are limited by elevations in the concentrations of creatine kinase [16]. Common side effects include neutropenia, anemia and loss of taste sensation [23, 24].

We have previously established a human small intestine NET cell line (GOT1) derived from a surgically removed liver metastasis [25]. The GOT1 cells have retained characteristic properties of NETs, such as expression of SSTR2 and SSTR5, and can be successfully xenotransplanted to nude mice [26]. In addition, it has previously been shown that ^177^Lu-octreotate induces cell cycle arrest, apoptosis and dose dependent tumor volume reduction in GOT1 tumors [27–29].

Considering the promising results of both Hh pathway inhibitors and ^177^Lu-octreotate in NET model systems [19, 22, 30], we hypothesized that inhibition of hedgehog signaling in NETs would increase the efficacy of ^177^Lu-octreotate treatment. The aim of this study was to test this hypothesis by investigating the therapeutic effects of combined treatment with the Hh inhibitor sonidegib and ^177^Lu-octreotate, compared with those of the two monotherapies consisting of either sonidegib or ^177^Lu-octreotate, in GOT1 human small intestine NETs in nude mice.

## Methods

### Tumor and animal model

GOT1 tumor tissue samples were transplanted s.c. in the neck of 4-week-old female BALB/c nude mice (CAnN.Cg-Foxn1nu/Crl, Charles River, Japan and Germany) as previously described [31]. During transplantation, animals were anesthetized using i.p. injection of Domitor® vet. (1 mg/ml injection solution, Orion Pharma Animal Health, Sweden) and Ketaminol® vet. (50 mg/ml injection solution, Intervet AB, Sweden). Antisedan (5 mg/ml injection solution, Orion Pharma Animal Health, Sweden) was injected i.p. after transplantation as antidote. Drinking water and autoclaved food were provided ad libitum.

### Pharmaceuticals

Sonidegib was purchased from Active Biochemicals Co., Limited (Hong Kong, China) and dissolved in DMSO as per manufacturer’s instructions.

^177^LuCl_3_ and [DOTA^0^, Tyr^3^]-octreotate were purchased from the Nuclear Research & Consultancy Group (IDB Holland, the Netherlands). Preparation and radiolabeling were conducted per the manufacturer’s instructions. Instant thin layer chromatography (ITLCTM SG, PALL Corporation, USA) was used for quality control, with the mobile phase consisting of 0.1 M sodium citrate (pH 5; VWR International AB, Sweden). The fraction of peptide-bound ^177^Lu was >98% and the specific activity was approximately 26 MBq/μg octreotate. Saline solution was used to dilute the ^177^Lu-octreotate stock solution to the desired activity concentration for administration. ^177^Lu activity in syringes was measured before and after injection using a well-type ionization chamber (CRC-15R; Capintec, IA, USA).

### Study design

In total, 21 GOT1 tumor-bearing mice were included in the study (Table 1). Tumor volumes varied between 0.1 and 2.5 ml (measured with slide calipers) at the start of experiments and an effort was made to obtain similar tumor size distributions in all experimental groups. Ten animals were divided into two treatment groups (*n* = 5/group). One group was treated with sonidegib (80 mg/kg body weight twice a week via oral gavage), while another group received both sonidegib (following the same treatment schedules as the monotherapy group) and an injection of 30 MBq ^177^Lu-octreotate (a non-curative treatment) into the tail vein. Tumor growth in the treatment groups was compared with that of animals receiving 30 MBq ^177^Lu-octreotate monotherapy (*n* = 5) and control animals injected with saline solution (*n* = 6), which have been characterized in a previous study [29]. During the study period, tumor size measurements were performed twice-weekly using digital slide calipers. Animals were killed 41 days after treatment start using i.p. injection of Pentobarbitalnatrium vet. (60 mg/ml, Apotek Produktion & Laboratorier AB, Sweden), followed by cardiac puncture. Tumor tissue samples were excised and instantly frozen in liquid nitrogen for gene expression analysis.

**Table 1:** Number of GOT1-bearing mice used in each analysis after treatment with sonidegib, ^177^Lu-octreotate, or a combination of both pharmaceuticals, and in control animals

	Sonidegib	^177^Lu-octreotate	Sonidegib +^177^Lu-octreotate	Control
Tumor volume measurements	5	5^a^	5	6^a^
Dosimetric calculations	-	5	5	-
Gene expression analysis	3	3	3	3^a^
Protein expression analysis	3	3	3	3

^a^Data has been characterized in a previous publication [29]

### Dosimetry

The mean absorbed dose, *D*(*r*_*T*_, *T*_*D*_), to the target tissue, *r*_*T*_, was calculated according to the Medical Internal Radiation Dose Committee (MIRD) pamphlet 21 formalism [32]:

$$ D\left({r}_T,{T}_D\right)=\frac{\tilde{A}\left({r}_S,{T}_D\right){\sum}_i{E}_i{Y}_i\phi \left({r}_T\leftarrow {r}_S,{E}_i,{T}_D\right)}{M\left({r}_T,{T}_D\right)}, $$

where $\tilde{A}\left({r}_S,{T}_D\right)$ is the time-integrated activity in source tissue, *r*_*S*_, over dose-integration period, *T*_*D*_ $\left(\tilde{\mathrm{A}}={\int}_0^{T_D}A\left({r}_s,t\right)\  dt\right)$, and *M*(*r*_*T*_, *T*_*D*_) is the mass of the target tissue, *r*_*T*_. $\tilde{A}$ values for ^177^Lu activity were determined in the tumor samples using activity concentration data presented by Dalmo et al. using GOT1 tumor samples after injection of 15 MBq ^177^Lu-octreotate [29]. The mean energy emitted per nuclear decay *i*, ∑_*i*_E_*i*_Y_*i*_, was approximated to 147.9 keV/decay [33], including β-particles, Auger and conversion electrons. The absorbed fraction, *ϕ*(*r*_*T*_ ← *r*_*S*_, *E*_*i*_, *T*_*D*_), was set to 1 for all tumors, and *r*_*T*_ was set to be the same as *r*_*S*_ in all calculations.

### RNA extraction and analysis

Gene expression microarray analysis was performed using RNA from three tumor samples per group (treated and control, for a total of 12 animals). Frozen tumor tissue was homogenized with the TissueLyser LT (Qiagen, Hilden, Germany) and total RNA was extracted using the RNeasy Lipid Tissue Mini Kit (Qiagen, Hilden, Germany) per the manufacturer’s instructions.

RNA concentration and purity were determined using an ND-1000 Spectrophotometer (NanoDrop Technologies, Wilmington, DE, USA). RNA integrity was validated with the RNA 6000 Nano LabChip Kit and Agilent 2100 Bioanalyzer (Agilent Technologies, Palo Alto, CA, USA). RNA integrity number (RIN) values higher than 8.1 were used in the present investigation.

Hybridization of the RNA samples was performed at Swegene Center for Integrative Biology (SCIBLU, Lund University, Sweden) on Illumina HumanHT-12 v4 Whole-Genome Expression BeadChips (Illumina, San Diego, CA, USA), containing 47,231 probes per array. The beadchips were analyzed using Illumina iScan N240 microarray scanner (Illumina, San Diego, CA, USA).

### Western blot

Western blot was carried out to analyze activation-states of the Hh- and PI3K/AKT/mTOR pathways. Tumor tissue samples from the same animals used in the gene expression analysis were homogenized in RIPA Lysis and Extraction Buffer (Thermo Scientific) using the TissueLyser LT (Qiagen) and Bioruptor® (Diagenode). Cell debris was removed by centrifugation and the protein extract was stored at −20 °C. Protein extracts (100 μg) were run on SDS-PAGE using Mini-PROTEAN® TGX™ Precast Gels (Bio-Rad) and transferred to nitrocellulose membranes using the Trans-Blot® Turbo™ Transfer System (Bio-Rad). Antibodies specific to GLI1 (ab151796, Abcam), GLI2 (LS-C313075, LifeSpan BioSciences), S6 (#2217, Cell Signaling Technology), AKT (#9272, Cell Signaling Technology), p-AKT (#9271, Cell Signaling Technology) and GAPDH (ab9485, Abcam, used as control) were detected using Amersham ECL Rabbit IgG (NA934VS, GE Healthcare Life Sciences). SuperSignal® West Femto Maximum Sensitivity Substrate (Thermo Scientific) was used for detection and digitalized images were acquired using Fujifilm Luminescent Image Analyzer LAS-1000 (Fujifilm, Tokyo, Japan).

### Data processing and statistical analysis

All tumor volume measurements for each group were expressed as mean value and standard deviation (SD). Student’s t-test was used to compare data between groups using a two-tailed unpaired t-test, and *p* < 0.05 was considered statistically significant.

In the transcriptional analysis, data pre-processing and quantile normalization were performed on the raw signal intensities using the web-based BioArray Software Environment (BASE) system. Differentially expressed transcripts (between experimental groups) were identified using Nexus Expression 3.0 (BioDiscovery, El Segundo, CA, USA) as previously described [34, 35]. Transcripts with altered expression ≥1.5 fold (|log_2_-ratio| ≥ 0.58) and Benjamini-Hochberg-adjusted *p*-value < 0.01 were considered significantly regulated compared with untreated controls (hereafter referred to as *regulated*).

Analysis of affected canonical pathways related to human cancer [36] and upstream regulators was conducted using the Ingenuity Pathway Analysis (IPA) software (Ingenuity Systems, Redwood City, USA). The *p*-value of overlap between the experimental data and the Ingenuity knowledge base was calculated with Fisher’s exact test (significance threshold at *p* < 0.05). The z-score was used to determine the activation state of the upstream regulators; z > 2 indicates activation, while z < −2 indicates inhibition. The Gene Ontology database was used for analysis of regulated transcripts associated with cell death and cell cycle regulation (significance threshold at *p* < 0.05 using a modified Fisher’s exact test) [34].

## Results

### Anti-tumor effect of sonidegib or ^177^Lu-octreotate monotherapy on GOT1 tumors

**Fig. 1:**
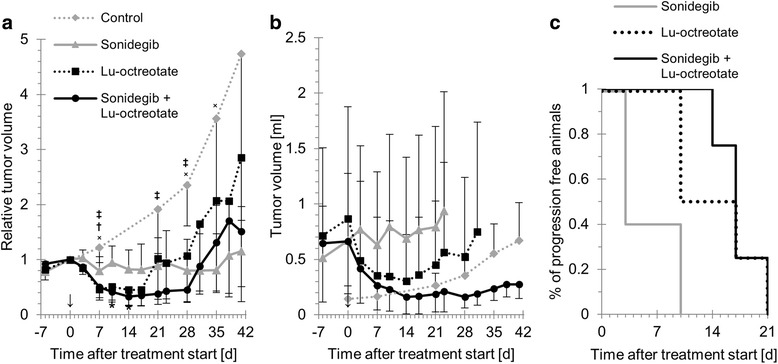
Anti-tumor effects of sonidegib, ^177^Lu-octreotate and combination treatment on GOT1 tumors in nude mice. **a** mean relative tumor volume versus time for controls and animals treated with sonidegib, ^177^Lu-octreotate, or a combination of both. **b** mean tumor volume versus time, until the first animal had to be killed on account of too high tumor burden, in each treatment group and controls. **c** percentage of animals in each treatment group without tumor progression. Error bars indicate SD. x, †, ‡ and * indicate time points with statistically significant differences between sonidegib and control, ^177^Lu-octreotate and control, combination and control, and combination and sonidegib treatment groups, respectively (Student’s t-test, *p* < 0.05). ↓ indicates the time for treatment start

Sonidegib monotherapy resulted in significant inhibition of tumor growth (Fig. 1a-b; minimum and maximum mean relative volumes 0.80 (SD = 0.25) and 1.2 (SD = 0.8), found at 7 and 41 d after treatment start, respectively). Statistically significant differences in mean relative volume between sonidegib-treated animals and controls were found at 7, 21, 28 and 35 d after treatment start.

The mean absorbed dose to the tumors receiving ^177^Lu-octreotate was 8 Gy at infinity time. Mean tumor volume relative to the day of injection was reduced in the group treated with ^177^Lu-octreotate monotherapy (Fig. 1a-b). The minimum relative tumor volume (mean = 0.45, SD = 0.29) was reached 14 d after injection. The mean relative tumor volume was below 1 from 3 d to 17 d after injection, after which the tumors began to regrow, resulting in a relative tumor volume of 2.9 at the end of the study. There was a statistically significant difference in relative tumor volumes between animals treated with ^177^Lu-octreotate and non-treated controls at 7 d after injection.

### Sonidegib enhances the anti-tumor effects of ^177^Lu-octreotate on GOT1 tumors

Combination treatment with sonidegib and ^177^Lu-octreotate caused a reduction in mean relative tumor volume (Fig. 1a). The minimum relative tumor volume for tumors receiving the combination therapy was lower than in either monotherapy group (mean = 0.33, SD = 0.16 at 14 d after injection). The mean tumor volume in the group treated with a combination of sonidegib and ^177^Lu-octreotate was also reduced after treatment, and showed the lowest values at all measurement time points after treatment start (Fig. 1b). Furthermore, the combination therapy group had a prolonged time to progression, i.e. time from treatment start to progression of first tumor in the treatment group (Fig. 1c), and the mean tumor volume at study end was smaller compared with the other groups (Fig. 1b). No symptoms of toxic effects were observed in the animals of either group. There was a statistically significant difference between relative tumor volume in animals treated with a combination of sonidegib and ^177^Lu-octreotate, and non-treated controls at 7, 21 and 28 d after treatment start. In addition, a statistically significant difference between combination treatment and sonidegib monotherapy was found at 10 and 14 d after treatment start.

### Different transcriptional responses in GOT1 tumors after monotherapies and combined treatment with sonidegib and ^177^Lu-octreotate

**Fig. 2:**
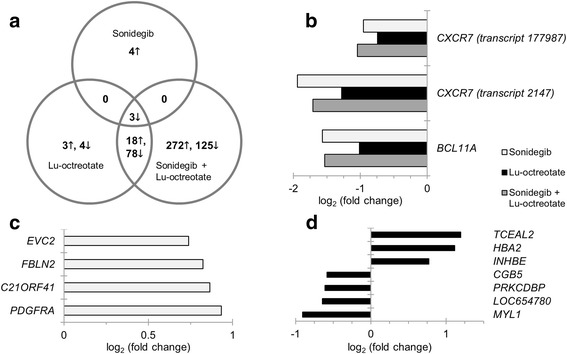
Distribution of differentially expressed genes in GOT1 tumors after sonidegib, ^177^Lu-octreotate and combination treatment. Differentially expressed transcripts in tumors treated with sonidegib, ^177^Lu-octreotate, or a combination of both pharmaceutical agents (treated vs. control). **a**: Venn diagram showing the distribution of up- (**↓**) and downregulated (**↑**) transcripts. Four, seven and 397 transcripts were uniquely regulated after sonidegib, ^177^Lu-octreotate and combination treatment, respectively; 96 transcripts were regulated after both ^177^Lu-octreotate and combination treatment; three transcripts were regulated in all treatment groups. **b**, **c** and **d**: Regulation patterns for the three commonly regulated transcripts and the four and seven transcripts uniquely regulated after sonidegib and ^177^Lu-octreotate treatment, respectively, with corresponding Illumina probe IDs. Transcripts with adjusted *p*-value < 0.01 and |log_2_(fold change)| ≥ 0.58 were considered significantly regulated. Up- and downregulation is indicated by positive and negative values, respectively

Genome-wide transcriptional microarray analysis of total RNA revealed diversity in gene regulation between treatment groups, compared with non-treated controls; the distribution and total number of regulated genes differed widely between treatment groups (Fig. 2). Seven, 106 and 496 transcripts were significantly regulated in the sonidegib, ^177^Lu-octreotate, and combination treatment groups, respectively. Four, seven and 397 transcripts were uniquely regulated in each group, while two genes (corresponding to three transcripts), *BCL11A* (involved in negative p53-regulation) and *CXCR7* (encoding a chemokine receptor), were regulated in all treatment groups (Fig. 2b-d). The *EVC2* and *PDGFRA* genes involved in the Hh pathway were among the four uniquely regulated transcripts in the sonidegib treatment group.

### Differential effects on cell signaling in GOT1 tumors after sonidegib and ^177^Lu-octreotate

**Fig. 3:**
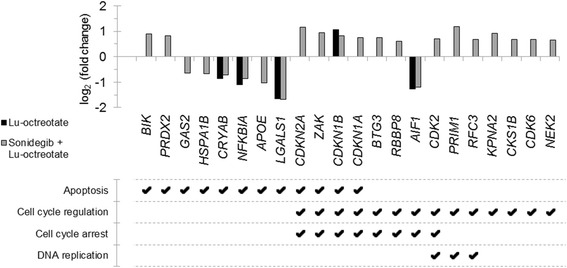
Expression of genes associated to cell death and/or cell cycle regulation in GOT1 tumors after ^177^Lu-octreotate and combination treatment. Differential expression (treated vs. control) of transcripts associated with cell death- and/or cell cycle regulation-processes in tumor samples from animals treated with ^177^Lu-octreotate (black), or a combination of sonidegib and ^177^Lu-octreotate (gray). No significant association with cell death or cell cycle regulation was found in tumor samples from animals treated with sonidegib monotherapy. Transcripts with |log_2_(fold change)| ≥ 0.58 and adjusted *p* < 0.01 were considered significantly regulated, association to biological processes was performed using the Gene Ontology database (threshold *p* < 0.05). Up- and downregulation is indicated by positive and negative values, respectively

IPA analysis based on differentially expressed genes predicted that four upstream regulators (CTBP1, PRKCA, HIC1 and SUZ12) were significantly affected in all treatment groups; five affected upstream regulators were commonly detected in the sonidegib and ^177^Lu-octreotate treatment groups and three were shared between the sonidegib and combination treatment groups (Table 2A). An analysis of the activation state of the upstream regulators resulted in prediction of two significantly activated (FSH and Lh) and one significantly inhibited (INS) upstream regulators (Table 2B). Gene Ontology analysis revealed regulation of several genes related to apoptotic cell death and cell cycle regulation (including several related to cell cycle arrest or DNA replication) in the ^177^Lu-octreotate and combination therapy groups (Fig. 3), but none in the sonidegib group.

**Table 2:** Predicted upstream regulators in GOT1 tumors after sonidegib, ^177^Lu-octreotate, or a combination of both treatments

Upstream Regulator	Molecule Type		*p*-value:		Targets from data
		Sonidegib	^177^Lu-octreotate	Combination	
A					
SUZ12	Enzyme	2.0·10^−2^	4.6·10^−3^	1.3·10^−4^	UP: *CDKN2A,* *HSD17B11, LITAF* DOWN: *CXCR7, KCNA1, RBMS1*
CTBP1	Enzyme	2.4·10^−3^	3.9·10^−2^	1.3·10^−2^	UP: *CDKN1A* DOWN: *CXCR7*
PRKCA	Kinase	1.2·10^−2^	1.9·10^−2^	2.5·10^−3^	DOWN: *BCL11A, EGR1,* *ETS1, HSPA1B, PVR*
HIC1	Transcription regulator	1.6·10^−2^	2.9·10^−2^	3.3·10^−2^	UP: *CDO1* DOWN: *CXCR7, ID4, KCNJ6*
miR-292b-5p	Mature microRNA	1.1·10^−3^	1.8·10^−2^	-	DOWN: *BCL11A*
LIN28B	Other	1.3·10^−3^	2.2·10^−2^	-	DOWN: *BCL11A*
CHD4	Enzyme	2.7·10^−3^	4.4·10^−2^	-	DOWN: *BCL11A*
EZH2	Transcription regulator	2.4·10^−2^	8.1·10^−3^	-	DOWN: *CXCR7, KCNA1, NFKBIA*
MAPK1	Kinase	4.2·10^−2^	3.5·10^−2^	-	UP: *HBA2* DOWN: *BCL11A, CTNNA2*
Histone h3	Group	3.9·10^−2^	-	2.1·10^−4^	UP: *BTG3, CDKN1A, CDKN2A, ENO3, PCNA, RGS10, STOM* DOWN: *CXCR7, NFKBIA, SOD2*
Ctbp	Group	3.2·10^−3^	-	2.3·10^−2^	UP: *CDKN2A* DOWN: *CXCR7*
ETS1	Transcription regulator	1.7·10^−2^	-	4.2·10^−2^	DOWN: *BCL11A, CTGF, HSPA1B, PVR*
B					
Upstream Regulator	Molecule Type	z	*p*-value	Targets from data	
INS	Other	−2.1	2.6·10^−4^	UP: *CBS, LPL* DOWN: *EGR1, OGT, SREBF1*	
FSH	Complex	2.2	7.5·10^−3^	UP: *DLX5, GNAS, PMAIP1, RGS16, SC4MOL* DOWN: *BCL11A, DUSP3, FILIP1L, FLNC, ILK, ITGA3, TPM2*	
Lh	Complex	2.2	2.4·10^−2^	UP: *GNAS, PMAIP1, RGS16, SC4MOL* DOWN: *DUSP3, FLNC, ILK, ITGA3, TPM2*	

A) Upstream regulators with statistically significant *p*-values (*p* < 0.05) in at least two treatment groups. B) Upstream regulators with activation |z-score| > 2, identified in tumor samples treated with a combination of sonidegib and ^177^Lu-octreotate. No upstream regulators with |z-score| > 2 were found in either monotherapy group. z > 2 indicates activation, z < −2 indicates inhibition. UP and DOWN indicate up- and downregulation, respectively

### Impact on cancer-related signaling pathways in GOT1 tumors after sonidegib and ^177^Lu-octreotate

The gene expression data was further analyzed by IPA. The affected signaling pathways in GOT1 tumors after treatment were identified by IPA analysis using differentially regulated genes. The signaling pathways in human cancer generated by IPA are presented in Table 3. The NF-ΚB signaling pathway was significantly affected in the group treated with sonidegib monotherapy – owing to the unique upregulation of the *PDGFRA* gene (encoding the platelet-derived growth factor receptor, alpha polypeptide). Interestingly, the gene encoding the ligand for the PDGFRA receptor (*PDGFA*) was downregulated in both the ^177^Lu-octreotate and combination therapy groups (log_2_-ratios of −1.5 and −1.7, respectively). The Wnt/β-catenin signaling pathway was significantly affected in both the ^177^Lu-octreotate and combination therapy groups, where the *SOX2*, *TLE4*, and *WNT11* genes were downregulated in both groups. However, a larger number of genes in the Wnt/β-catenin pathway were affected in the combination therapy group. The PI3K/AKT/mTOR-, G-protein coupled receptor-, and Notch-signaling pathways were also affected in the combination therapy group.

**Table 3:** Affected cancer-related canonical pathways in GOT1 tumors after sonidegib, ^177^Lu-octreotate, or combination of both treatments

Canonical pathway	Treatment	*p*-value	Targets from data
Wnt/β-catenin	Sonidegib	-	-
signaling	^177^Lu-octreotate	0.045	DOWN: *SOX2, TLE4, WNT11*
	Combination	0.003	UP: *CDKN2A, PPP2R2C* DOWN: *FZD9, GNAQ, ILK, SOX2, TCF4, TGFB3, TLE4, WNT11*
PI3K/AKT/mTOR signaling	Sonidegib	-	-
	^177^Lu-octreotate	0.110	UP: *CDKN1B* DOWN: *NFKBIA*
	Combination	0.013	UP: *CDKN1A, CDKN1B, PPP2R2C, PTEN* DOWN: *ILK, ITGA3, NFKBIA*
G-protein coupled	Sonidegib	-	-
receptor signaling	^177^Lu-octreotate	-	-
	Combination	0.043	UP: *GNAQ, GNAS, GRM8, PDE5A, RGS10, RGS16* DOWN: *ADCY3, CAMK2B, GNAI2, NFKBIA*
Notch signaling	Sonidegib	-	-
	^177^Lu-octreotate	-	-
	Combination	0.043	DOWN: *DLL1, LFNG, PSENEN*
NF-ΚB signaling	Sonidegib	0.048	UP: *PDGFRA*
	^177^Lu-octreotate	0.181	DOWN: *GHR, NFKBIA*
	Combination	-	-

Selection of Ingenuity canonical pathways (with statistical significance (*p* < 0.05) for at least one treatment group) related to human cancer [36], enriched by differentially expressed genes in each treatment group. UP and DOWN indicate up- and downregulation, respectively

### Protein expression analysis reveals Hh-activation downstream of SMO, and activation of PI3K/AKT/mTOR

**Fig. 4:**
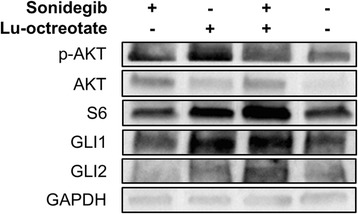
Expression of proteins in the Hh- and PI3K/AKT/mTOR pathways in GOT1 tumors after sonidegib, ^177^Lu-octreotate and combination treatment. Expression of Hh-related (GLI1, GLI2) and PI3K/AKT/mTOR-related (AKT, p-AKT, S6) proteins in tumors treated with sonidegib, ^177^Lu-octreotate, or a combination of both pharmaceutical agents, and untreated controls, measured using western blot analysis. Representative immunoblots from each group are shown. + and - indicates treated and untreated, respectively

Western blotting showed increased amounts of GLI1 in tumors from animals treated with ^177^Lu-octreotate monotherapy and combination treatment, and increased amounts of GLI2 in tumors from the combination therapy group, compared with controls (Fig. 4). This indicates an activation of the Hh pathway in these tumors. Protein levels of AKT and p-AKT were elevated in all three treatment groups, while S6 was elevated in tumors from the ^177^Lu-octreotate monotherapy and combination treatment groups (Fig. 4).

## Discussion

PRRT using ^177^Lu-octreotate is a promising treatment option for patients with NETs, with longer progression-free survival and higher response rates than alternative treatments [7]. However, due to dose limiting risk organs, curative treatment is still rare. This study describes the first combination treatment for neuroendocrine tumors with Hh pathway inhibition and PRRT. We show that, given as monotherapy, both sonidegib and ^177^Lu-octreotate have anti-tumor effects on GOT1 tumors in nude mice, with sonidegib resulting in inhibition of tumor growth over time and ^177^Lu-octreotate resulting in initial tumor volume regression followed by regrowth, in agreement with previous studies [16, 22, 27, 28]. The initial tumor volume response in the animals treated with a combination of sonidegib and ^177^Lu-octreotate mimicked that of the ^177^Lu-octreotate monotherapy. However, the time to progression was longer in the combination therapy group, resulting in the lowest mean tumor volume at the time of study end. This indicates a potential benefit when using Hh inhibitors in combination with ^177^Lu-octreotate for treatment of small intestine neuroendocrine tumors. However, further studies on the difference in adverse effects between different treatment schedules are needed, especially concerning adverse effects on risk organs (e.g. kidneys and bone marrow).

The tumor absorbed dose in animals receiving 30 MBq ^177^Lu-octreotate was estimated to 8 Gy at infinity time, assuming homogeneous activity distribution and based on the biokinetics of 15 MBq ^177^Lu-octreotate [29]. However, saturation of the SSTR is an issue that must be considered when using radiolabeled somatostatin analogs and it is possible that the 30 MBq used in this study may have resulted in a lower mean absorbed dose to the tumor [37]. In the present study, it was not possible to define the level of potential saturation.

A substantially higher number of genes were regulated in the combination therapy group compared with the two monotherapy groups. *CXCR7* and *BCL11A* were regulated in all groups. The *CXCR7* gene has been studied in human breast cancer models, where treatment with a CXCR7 antagonist has been shown to delay tumor growth and increase survival rates [38]. *CXCR7* has also been identified as a possible downstream target of Hh pathway members GLI1 and GLI2 [14]. The *BCL11A* gene was downregulated in all three treatment groups. It negatively regulates p53 by directly regulating the *BCL2*, *BCL-* *x* *L*, *MDM2* and *MDM4* genes. Consequently, downregulation of the *BCL11A* gene might result in apoptotic and proliferative defects [39]. Ninety-six transcripts were regulated in both the ^177^Lu-octreotate and combination therapy groups. In similarity with the two commonly regulated genes, these were all regulated in the same direction (i.e either downregulated in both groups, or upregulated in both groups), and the expression levels were roughly similar (see Additional file 1). These results indicate that the combination therapy increased the diversity of transcriptional regulation, while having minor effects on the extent of regulation.

Several of the uniquely regulated genes have been associated with Hh signaling, namely the *EVC2* and *PDGFRA* genes in the sonidegib group and the *GNAS* gene in the combination treatment group. The *EVC2* gene has been identified as a tissue-specific regulator of Hh signaling: The EVC2 protein binds to SMO after it accumulates in cilia in response to Hh ligands, and upregulation of the *EVC2* gene can activate the Hh pathway downstream of SMO, but upstream of GLI transcription factors [12]. The *PDGFRA* gene is a transcriptional target of GLI1, and downregulation of the *PDGFRA* gene has previously been associated with decreased GLI1 levels despite Hh pathway activation [40]. In the present study, upregulation of the *EVC2* and *PDGFRA* genes in the sonidegib group may therefore correspond to activation of the Hh pathway downstream of SMO, countering the effect of the SMO antagonist.

Several of the regulated genes were associated with apoptotic cell death and cell cycle regulation. Among these, the *CDK2* and *CDK6* genes involved in cell cycle checkpoint activity have been found to be activated by GLI1, independent of SMO activation status [41, 42]. In addition, several genes involved in the TP53-signaling pathway were regulated in the present study, corresponding to both growth arrest (e.g. *BTG3*, *CDK6, CDKN1A (p21)*, *CDKN1B (p27)*, and *CDKN2A (p16)*) and apoptosis (e.g. *APOE* and *BIK*) [43–47].

The IPA pathway analysis resulted in the prediction of several cancer-related signaling pathways. The Wnt/β-catenin signaling is important in regulating cancer cell invasiveness, and has been found to be implicated in the acquisition of radioresistance and radiation-induced cell invasion in glioblastomas [48]. Downregulation of several key components of the Wnt/β-catenin pathway (e.g. *FZD9* and *WNT11*) in the combination therapy group suggests that evasive radioresistance may be reduced following this treatment regimen. G-protein coupled receptor signaling was also found to be affected by the combination treatment. Out of the molecular targets for the treatments used in the present study, SSTR are G-protein coupled receptors, and SMO has been classified as a G-protein coupled receptor or a G-protein coupled receptor-like receptor. G-protein coupled receptor signaling is a major factor in many cellular functions in cancers [49]. These diverse biological functions complicate an interpretation of the predicted effect on G-protein coupled receptor signaling. However, the unique upregulation of the *GNAS* gene in the combination therapy group indicates a possible inhibition of the Hh pathway. The *GNAS* gene encodes the heterotrimeric G_s_-protein α subunit (Gα_s_), which transmits various G-protein coupled receptor signals regulating, e.g. cell growth and survival. Previous in vivo studies have shown that the *GNAS* gene can act as a tumor suppressor in Hh-driven medulloblastomas [50]. The Notch signaling pathway was also predicted to be affected in the group receiving a combination of sonidegib and ^177^Lu-octreotate. Notch has a direct role in DNA damage response and Notch inhibitors have been considered for treatment of various cancers in combination with radiotherapy [51]. Inhibition of Notch has been shown to prevent upregulation of Notch ligands, e.g. *DLL1*, after radiotherapy in breast cancer cells, and the downregulation of *DLL1* in the combination therapy group in the present study may indicate a possible explanation of the mechanism involved in the enhanced anti-tumor effects in this treatment group [52].

The PI3K/AKT/mTOR signaling pathway was predicted to be activated in the combination therapy group. This pathway has previously been recognized as a possible candidate for combination therapy with PRRT, since the mTOR signaling pathway is often upregulated in NETs and the mTOR inhibitor everolimus has shown promising anti-NET results [9]. However, a previous study found that a combination treatment with everolimus and ^177^Lu-octreotate promotes metastasis in a pancreatic NET model in rats [9]. The mTOR target S6 (a serine/threonine kinase) has previously been shown to activate GLI1 in multiple cancer types, independent of SMO, indicating a crosstalk between the PI3K/AKT/mTOR- and Hh pathways [16, 53]. Furthermore, a combination of PI3K/AKT/mTOR- and Hh inhibitors have been shown to have more potent anti-tumor effects than either monotherapy [53, 54]. Our western blot data showed elevated levels of GLI1, GLI2 and S6 in both the ^177^Lu-octerotate monotherapy and combination therapy groups. This suggests that ^177^Lu-octerotate may lead to SMO-independent Hh-activation via the PI3K/AKT/mTOR pathway, indicating a possibility for further increased therapeutic results from a triple-combination of ^177^Lu-octerotate, sonidegib and a PI3K/AKT/mTOR inhibitor.

## Conclusions

In summary, combination therapy of GOT1 tumors in nude mice using sonidegib and ^177^Lu-octreotate resulted in a profound reduction in tumor volume shortly after treatment start, similar to the effect of ^177^Lu-octreotate monotherapy. In contrast to the ^177^Lu-octreotate monotherapy, a prolonged time to progression (tumor regrowth) was observed in the combination therapy group. These results show that combination therapy using sonidegib and ^177^Lu-octreotate could be beneficial to patients with NE-tumors, but further studies are needed to determine the optimal dose of sonidegib and ^177^Lu-octreotate, regarding anti-tumor and toxic effects.

Gene expression analysis revealed an interaction between sonidegib and ^177^Lu-octreotate, affecting several cancer-related signaling pathways (i.e. Wnt/β-catenin, PI3K/AKT/mTOR, G-protein coupled receptor, and Notch) not affected by either monotherapy. This may explain the underlying mechanisms of the enhanced anti-tumor effects from combination treatment with sonidegib and ^177^Lu-octreotate. Protein expression analysis indicated a possible PI3K/AKT/mTOR-dependent activation of GLI1 and GLI2, independent of SMO. This indicates that future studies of combination therapy using ^177^Lu-octerotate, sonidegib and a PI3K/AKT/mTOR inhibitor are warranted.

## Additional file

Additional file 1: Table S1. Regulation patterns for the 96 transcripts regulated both after ^177^Lu-octreotate monotherapy and after combination therapy with sonidegib and ^177^Lu-octreotate. (XLS 40 kb)

## Abbreviations

^177^Lu-octreotate: ^177^Lu-[DOTA^0^, Tyr^3^]-octreotate
BASE: BioArray Software Environment
Hh: Hedgehog
IPA: Ingenuity Pathway Analysis
MIRD: Medical Internal Radiation Dose Committee
NET: Neuroendocrine tumor
PRRT: Peptide receptor radionuclide therapy
RIN: RNA Integrity Number
SCIBLU: Swegene Centre for Integrative Biology at Lund University
SD: Standard deviation
SMO: Smoothened
SSTR: Somatostatin receptor
